# A Comparison of Neuropathic Pain Experiences Among Paralympic Versus Recreational Athletes with Spinal Cord Injury

**DOI:** 10.1186/s40798-023-00645-w

**Published:** 2023-12-09

**Authors:** Kendra R. Todd, Kenedy Olsen, Gail Hamamoto, Trevor J. Hirschfield, John L. K. Kramer, Kathleen A. Martin Ginis

**Affiliations:** 1 School of Health and Exercise Sciences, University of British Columbia, 3333 University Way, Kelowna, BC V1V1V7 Canada; 2 International Collaboration On Repair Discoveries (ICORD), Blusson Spinal Cord Centre (BSCC), University of British Columbia, Vancouver, Canada; 3 British Columbia Wheelchair Sports Association, Vancouver, Canada; 4 Wheelchair Rugby Canada, Ottawa, Canada; 5 Department of Anesthesiology, Pharmacology, and Therapeutics, University of British Columbia, Vancouver, Canada; 6 Department of Medicine, Division of Physical Medicine and Rehabilitation, University of British Columbia, Vancouver, Canada; 7 Centre for Chronic Disease Prevention and Management, University of British Columbia, Kelowna, BC Canada

**Keywords:** Chronic pain, Paralympic sport, Exercise, Disability, Spinal cord injury

## Abstract

**Background:**

Individuals with spinal cord injury (SCI) report high levels of neuropathic pain. Current treatment options are primarily pharmaceutical, despite their limited effectiveness. Exercise may reduce neuropathic pain among persons with SCI; however, the optimal dose of exercise required to elicit analgesic effects remains unknown. The purpose of this study was to compare neuropathic pain intensity, pain catastrophizing, use of coping strategies, and positive affect and well-being among Paralympic versus recreational athletes with SCI who experience chronic neuropathic pain. Forty-seven athletes with SCI (25 Paralympic, 27 recreational) completed the International SCI Pain Basic Data Set, Douleur Neuropathique-4, coping strategies questionnaire, pain catastrophizing scale, and SCI-quality of life assessment.

**Results:**

Paralympic athletes reported significantly greater neuropathic pain (*p* = 0.032) and positive affect and well-being (p = 0.047) than recreational athletes. No other comparisons were significant (*p*s > 0.09). Significant, medium-sized positive correlations were observed between neuropathic pain and total minutes of moderate-intensity exercise (*r* = 0.335, *p* = 0.023) and average minutes per day of moderate-intensity exercise (*r* = 0.375, *p* = 0.010) over the past week.

**Conclusions:**

The results suggest that frequent moderate- to high-intensity exercise may exacerbate neuropathic pain sensations for persons with SCI. Research should investigate psychosocial and physiological mechanisms by which exercise may influence neuropathic pain to explain how Paralympic athletes with SCI are able to continue exercising while maintaining positive affect despite neuropathic pain.

## Key Points

1. Paralympic athletes with SCI report significantly greater neuropathic pain intensity than recreational athletes with SCI, but also higher levels of positive affect and well-being.
2. Neither pain catastrophizing, pain coping, nor positive affect and well-being significantly moderated the relationship between athlete status (i.e., Paralympic or recreational athlete) and neuropathic pain intensity.
3. Significant, positive correlations were observed between total weekly moderate-intensity exercise and neuropathic pain. No significant correlations were observed between frequency of heavy-intensity exercise and neuropathic pain .

## Background

Approximately 58% of persons with a spinal cord injury (SCI) experience neuropathic pain [1, 2], with many reporting their pain to significantly impair functioning, increase disability and reduce quality of life [3, 4]. Allodynia (pain resulting from a non-noxious stimulus) and hyperalgesia (heightened response from a noxious stimulus) are common consequences of neuropathic pain [5], as are sensations such as burning, tingling or sharpness. Current treatment options for neuropathic pain are primarily pharmaceutical; however, in addition to causing debilitating side effects, pharmaceuticals result in just 50% pain reduction for only 30% of individuals with SCI [6]. There is a critical need to identify effective pain management techniques that are complementary to pharmaceuticals and align with the preferences and needs of individuals who experience SCI-neuropathic pain [7, 8].

The frequency, duration and intensity of exercise required to reduce neuropathic pain among adults with SCI (or in any clinical population that experiences neuropathic pain) is not yet known. To the best of our knowledge, only four published studies have examined the relationships between exercise and neuropathic pain among persons with SCI [9–12]. In a case series study, all six participants reported decreased neuropathic pain sensations following at least one of two bouts of moderate-intensity, community-based exercise performed within a single week [9]. In two experimental design studies, neuropathic pain decreased following a single bout of high-intensity aerobic exercise (arm crank ergometer or wheelchair propulsion) [10, 11]. And in an exercise training study, 10 weeks of high-intensity aerobic exercise (seated double poling arm ergometry), 3 times per week, led to decreased neuropathic pain for 4 of the 7 participants who experienced SCI neuropathic pain [12]. Together, these studies suggest that moderate- and high-intensity acute bouts of exercise can decrease neuropathic pain, as can high-intensity exercise performed over a couple of months. However, we do not know about the effects of habitual high-intensity exercise that is done at a high-frequency [HFHI (e.g., 5 sessions/week for 30 min at 80% VO_2_ max; [13])].

Although adults with SCI are typically considered a low-active population [14], Paralympic athletes with SCI routinely train at high exercise frequencies and intensities. For example, among wheelchair rugby athletes with SCI, within-game peak ratings of perceived exertion often exceed 16 (i.e., ‘very hard to extremely hard’ intensity; [15]). Paralympic athletes with SCI also frequently report chronic pain. Fifty percent reported moderate to severe musculoskeletal pain intensity every week over a 52-week observational period [16]. While these data suggest frequent, high-intensity exercise may be linked to *musculoskeletal* pain in athletes with SCI, we do not know if HFHI exercise is also associated with *neuropathic* pain in this population. The pervasive and negative impact of neuropathic pain on health and well-being [1], together with its resistance to common treatments [1, 17], highlight the need to better understand neuropathic pain experiences among Paralympic athletes with SCI.

The primary purpose of this study was to compare neuropathic pain experiences among Paralympic versus recreational athletes with SCI. Comparisons across these two groups will advance understanding of the association between neuropathic pain and longer-term participation in relatively higher versus lower intensities and frequencies of exercise. Given that preliminary data suggests higher-intensity exercise can reduce neuropathic pain in some adults with SCI [10–12], it was hypothesized that Paralympic athletes would experience significantly lower neuropathic pain than recreational athletes.

A second objective was to test whether pain catastrophizing, use of pain coping strategies, and levels of positive affect and well-being moderate the relationship between exercise and neuropathic pain intensity. Among able-bodied individuals, research has demonstrated that situational pain catastrophizing (e.g., hyper-focusing on pain) and negative mood states are associated with greater pain intensity following exercise [18]. Similarly, among persons with SCI, longitudinal studies have shown that greater pain catastrophizing can lead to greater pain intensity [19, 20]). For people with low back pain, greater use of pain coping strategies, such as distraction techniques, significantly moderated the relationship between exercise and pain intensity [21]. Based on these findings, it was hypothesized that athletes who have greater levels of positive affect and well-being, and athletes who use more pain coping strategies, would experience lower neuropathic pain intensity.

An exploratory objective was to examine the relationships between neuropathic pain intensity and the frequency and duration of moderate- and high-intensity exercise. Preliminary data on these relationships will be important for formulating hypotheses in future studies of the effects of different exercise prescriptions on SCI neuropathic pain. As only four published studies have examined the relationships between exercise and neuropathic pain among adults with SCI [9–12], and none of those studies compared different frequencies or durations of exercise, it was impossible to formulate a meaningful hypothesis with regard to the expected direction of the relationships.

## Methods

### Study Design

Our study objectives were addressed using a nonrandomized, quasi-experimental design. We compared neuropathic pain experiences between two groups of physically active individuals with SCI who reported chronic neuropathic pain: Paralympic athletes training for the 2021–22 Paralympic games at time of assessment and recreational athletes.

### Participants

Sample size was based on research among able-bodied individuals indicating that high-intensity exercise may lead to a clinically significant decrease (30%) in pain intensity, whereas moderate-intensity exercise may not [22]. Using a Fisher’s exact proportion test, G*Power 3.1.9.4 [23] estimated *n* = 44 (22 participants/group) provided 80% power (*α* = 0.05) to detect a 30% difference in neuropathic pain intensity among Paralympic versus recreational athletes with SCI.

Participant inclusion criteria were as follows: (1) > 18 years old; (2) incurred an SCI > 12 months ago at the third cervical level or below; (3) experience chronic SCI neuropathic pain; (4) able to read/write in English; (5) National Paralympic team member currently training for 2021 or 2022 Paralympic games (Paralympic athlete condition) OR routinely achieving the SCI scientific exercise guideline for fitness improvements: 20 min of moderate- to vigorous-intensity aerobic activity two times per week, and strength training two times per week, consisting of three sets of 8–10 repetitions of each exercise for each major muscle group (recreational athlete condition; [24]).

Participants were recruited worldwide between January-June, 2021 through social media, email, global parasport organizations and word-of-mouth. Fifty-five individuals volunteered to participate. After screening, forty-seven met the inclusion criteria (22 Paralympic athletes, 25 recreational athletes).

### Procedure

Eligible volunteers were sent an email with detailed study information and informed consent forms. After providing informed consent, participants were scheduled for a telephone call with the first author. The purpose of this call was for the researcher to administer the measures of pain and exercise. In preparation for this call, participants were emailed SCI-specific descriptions of moderate- and heavy-intensity exercise [25]. The telephone conversations were audio recorded, and the ISCIPBDS was transcribed verbatim by the second author. Afterward, participants were sent a personalized link to complete the remaining questionnaires online, by themselves, using the REDCAP® survey platform.

## Measures

### Neuropathic Pain

Neuropathic pain intensity was measured using the *International SCI Pain Basic Data Set* (ISCIPBDS v2.040; [26]). The ISCIPBDS is administered as a structured interview, such that each question is read to the participant as worded. The interview questions are used to collect data on the characteristics (e.g., intensity, location) of participants’ top three pain problems. Intensity is measured on a 10-point numerical rating scale (0 = no pain, 10 = pain as bad as you can imagine). Use of pain treatment(s) is measured using a dichotomous ‘yes or no’ question. The ISCIPBDS has shown acceptable reliability and validity in clinical and research settings [27] and is the internationally recommended method for collecting clinically relevant pain data in persons with SCI [26].

The correct classification of pain as neuropathic was corroborated using the Douleur Neuropathique-4 (DN-4). This is a diagnostic tool for determining if pain has a neuropathic component [28]. The DN-4 includes seven questions evaluating pain characteristics (e.g., burning, tingling, electric shocks), in addition to three questions to determine the presence of hypoesthesia or allodynia. Scores on the DN-4 can range from 0 to 10, with scores of ≥ 4 indicating the presence of neuropathy. The DN-4 has demonstrated acceptable psychometrics among persons with SCI [29].

### Exercise Participation

The Leisure Time Physical Activity Questionnaire for people with SCI (LTPAQ-SCI) is a SCI-specific self-report measure of the frequency, intensity and duration of exercise, sports, and other recreational physical activities [30]. Participants were asked to recall over the past 7 days, the (a) number of days, and (b) number of minutes on those days, that they spent engaging in mild-, moderate- and heavy-intensity exercise/sports/recreation. To aid valid classification of exercise intensity, before administering the questionnaire, the first author reviewed validated SCI-specific definitions of exercise intensity with the participant [25]. The LTPAQ-SCI has demonstrated validity and reliability among persons with SCI as a measure of minutes per week of leisure-time physical activity (LTPA) [30, 31]. Total number of minutes of moderate-intensity and heavy-intensity exercise performed over the previous 7 days was calculated by multiplying the number of days of activity by the number of minutes of activity at each of the two intensities.

### Potential Moderating Variables

#### Coping Strategies

The single-item coping strategies questionnaire (CSQ; [32]) was used to assess the frequency of using seven pain coping strategies: diverting attention, reinterpreting pain sensations, ignoring pain, praying and hoping, coping self-statements, increasing behavioral activities, and catastrophizing. Strategies were rated on a 6-point frequency scale ranging from 0 (never do that) to 6 (always do that when in pain). The CSQ has demonstrated validity in several different patient groups, including those with SCI [33]. The CSQ is scored (0–42) by summing responses to the seven strategies. A higher score indicates a greater use of pain coping strategies.

#### Pain Catastrophizing

The Pain Catastrophizing scale (PCS; [34]) assesses rumination, magnification, and helplessness. Participants were asked to reflect on past experiences when they perceived high levels of neuropathic pain and to indicate on a 5-point scale ranging from 0 (not at all) to 4 (all of the time) the degree to which they experienced each of 13 thoughts or feelings when experiencing neuropathic pain. The PCS has demonstrated acceptable reliability, validity, and internal consistency among persons with SCI [35]. The PCS is scored (0–52) as a sum of the responses to the 13 items. A higher score indicates greater levels of pain catastrophizing.

### Positive Affect and Well-Being

Positive affect and well-being (PAWB) were assessed using the SCI-Quality of Life (SCI-QOL) v1.0 measurement system PAWB [36], which consists of 8 SCI-specific items. Participants were asked to rate how frequently they felt PAWB on a 5-point scale ranging from 1 (never) to 5 (always). Items included “I felt confident,” “I had a sense of well-being,” “I was optimistic about the future,” and “I felt cheerful.” The total scores were calculated by summing item responses, with higher scores indicating greater PAWB.

### Engagement of Stakeholders in the Study

Consistent with an integrated knowledge translation approach [37], stakeholders and research end-users were involved throughout the research process. First, an Executive Director of a provincial wheelchair sports association and one Paralympic athlete with SCI and knowledge of neuropathic pain were consulted to ensure the research questions were relevant to the community. These individuals also helped to select and modify measures to ensure questions were easily comprehensible. In addition, several Paralympic and recreational athletes and provincial sport organizations helped with participant recruitment.

### Statistical Analyses

Independent samples t-tests were conducted to test differences in neuropathic pain intensity and potential moderating variables between Paralympic and recreational athletes. Moderation effects were tested using the *PROCESS* software macro [38]. Neuropathic pain intensity was specified as the dependent variable with athlete status specified as the independent variable and coping, catastrophizing and PAWB as the moderator(s). Separate moderation analyses were computed for each potential moderator. Significance was set at *p* < 0.05, and effect sizes were interpreted according to Cohen’s conventions (small = 0.20, medium = 0.50, large = 0.80; [39]).

Pearson’s correlation coefficients were computed to determine if days per week, minutes per day, and total minutes per week of moderate- and high-intensity exercise are related to neuropathic pain intensity. Assumptions of normality, linearity, and presence of outliers were tested and met. Two-tailed tests were used. Cohen’s conventions were used for interpreting the magnitude of the correlations (small = 0.1, medium = 0.3, large = 0.5; [39]). SPSS version 22.0 was used for all analyses.

## Results

### Participant Characteristics

The majority of participants (*n* = 47) had paraplegia (53%), experienced a traumatic SCI (94%), were manual wheelchair users (79%) and had neurologically incomplete injuries (64%). The average age of participants was 38 ± 11.8 years, and ranged between 20 and 67 years. Participants’ average years post SCI was 13.7 ± 10.0 years, and ranged from 1 to 42 years. Participants lived in North America, South America, Australia, Africa, Asia and Europe. Paralympic athletes were competing in para Nordic skiing, para alpine, para canoe, para ice hockey, para swimming, wheelchair tennis, para table tennis, wheelchair athletics, wheelchair basketball and wheelchair rugby. Paralympic athletes were significantly younger than recreational athletes (*p* < 0.001); however, age was unrelated to any of the study outcome measures (*r*s ranged from − 0.139 to 0.118, all *p*s > 0.351) so it was not included as a covariate in subsequent analyses. There were no significant differences between the two groups in terms of the proportion of male versus female participants or on SCI-relevant variables (years post-injury, level or completeness of injury).

All participants reported > 4 on the DN-4 (M = 6.76 ± 1.7) confirming all participants experienced neuropathic pain. Twenty-nine participants were using or receiving treatment for their neuropathic pain (e.g., pharmaceutical, cannabis). No significant difference was found between groups for the number of neuropathic pain treatments used (*p* = 0.127).

As expected, Paralympic athletes reported significantly more minutes per week of moderate-intensity (*p* = 0.004), and heavy-intensity (*p* = 0.002) exercise than recreational athletes. Paralympic athletes reported double the minutes of moderate-intensity exercise and nearly triple the minutes of heavy-intensity exercise than recreational athletes. Complete demographic data are presented in Table 1, and M ± SD for LTPAQ variables are presented in Table 2.

**Table 1:** Demographic information of the sample (*n* = 47)

Variable	Paralympic athletes (M ± SD) *n* = 22	Recreational athletes (M ± SD) *n* = 25
Age (years)	32.3 ± 6.1	43.6 ± 13.2
Years post injury	13.5 ± 7.9	13.9 ± 11.7
*Sex*		
Male	13	19
Female	9	6
*Level of injury*		
Tetraplegia	11	11
Paraplegia	10	15
*Injury severity*		
Complete	4	9
Incomplete	18	16
*AIS classification*		
AIS A	4	9
AIS B	6	4
AIS C	3	4
AIS D	1	2
Unsure		6
*Cause of SCI*		
Traumatic	20	25
Non-traumatic	2	0
*Ethnicity*		
White	18	22
Other	4	3
*Marital status*		
Single	10	11
Common law	4	1
Married	7	13
Divorced	1	0
*Highest level of education*		
High school	7	6
College	4	7
University	7	7
Postgraduate	4	4
Other	0	1
*Type of Paralympic sport*		
Cross-country skiing	1	
Para alpine	1	
Para Canoe	1	
Para ice hockey	2	
Para kayaking	1	
Para swimming	1	
Para tennis	3	
Table tennis	1	
Wheelchair athletics	1	
Wheelchair basketball	2	
Wheelchair rugby	8	

AIS: American Spinal Injury Association Impairment Scale. AIS A = no motor or sensory function below the level of injury; AIS B = sensory function, but no motor function below the level of injury; AIS C = sensory function, partial motor function preservation below the level of injury; AIS D = sensory function, at least half of key muscle function preserved below the level of injury

**Table 2:** Descriptive statistics for the study variables

Outcome variables	Paralympic athletes	Recreational athletes	*p* value
Neuropathic Pain Intensity	5.91* ± 1.69	4.80 ± 1.73	0.032
Coping Strategies Questionnaire	18.50 ± 7.24	18.60 ± 5.77	0.960
Pain Catastrophizing Scale	14.14 ± 7.60	14.68 ± 9.94	0.836
Positive Affect and Well-being	37.23* ± 5.74	34.52 ± 4.35	0.047
Days/week moderate-intensity exercise	3.50 ± 2.09	3.72 ± 1.89	0.706
Average minutes/day moderate-intensity exercise	102.50 ± 69.23	49.00 ± 25.25	< 0.001
Total moderate-intensity exercise/week (mins)	421.60 ± 382.84	178.20 ± 105.35	0.004
Days/week heavy-intensity exercise	4.00 ± 1.60	2.28 ± 1.67	< 0.001
Average minutes/day heavy-intensity exercise	71.64 ± 51.19	37.60 ± 28.80	0.007
Total heavy-intensity exercise/week (mins)	300.23 ± 260.40	110.60 ± 131.71	0.002

Results are presented as M ± SD; * = significant at *p* < 0.05

### Main Analyses

Contrary to hypothesis, Paralympic athletes reported significantly greater neuropathic pain intensity (M = 5.91 ± 1.69) than recreational athletes (M = 4.80 ± 1.73, *t* [45] = 2.22, *p* = 0.032; ES = 0.74). However, Paralympic athletes reported experiencing significantly greater levels of positive affect and well-being (37.23 ± 5.74) than recreational athletes (34.52 ± 4.35, *t* [45] = 1.84, *p* = 0.047; ES = 0.536). No significant differences were observed between Paralympic and recreational athletes for reported use of coping strategies (*t* [45] = − 0.053, *p* = 0.958) or pain catastrophizing (*t* [45] = − 0.208, *p* = 0.836). Table 2 presents M ± SD for all study variables.

### Moderation Analyses

Results of the *PROCESS* analyses [38] indicated that neither pain catastrophizing, pain coping, nor positive affect and well-being, significantly moderated the relationship between athlete status (i.e., Paralympic or recreational athlete) and neuropathic pain intensity (*p*s > 0.336).

### Correlations Between Neuropathic Pain Intensity and Exercise

Significant, medium-sized positive correlations were observed between neuropathic pain intensity and total minutes of moderate-intensity exercise (*r* = 0.335, *p* = 0.023) and min/day of moderate-intensity exercise (*r* = 0.375, *p* = 0.010) over the past 7 days. Pain intensity was not significantly correlated with total minutes of heavy-intensity exercise (*r* = 0.005, *p* = 0.98) or min/day of heavy-intensity exercise (*r* = − 0.048, *p* = 0.75). See Table 3 for the full correlation matrix.

**Table 3:** Correlation matrix showing Pearson correlation coefficients for neuropathic pain intensity and measures of exercise

Measure	Neuropathic pain intensity	Moderate exercise (days/week)	Moderate exercise (min/session)	Moderate exercise (min/week)	Heavy exercise (days/week)	Heavy exercise (min/session)	Heavy exercise (min/week)
Neuropathic pain intensity	–						
Moderate exercise (days/week)	0.241	–					
Moderate exercise (min/day)	0.375**	0.222	–				
Moderate exercise (min/week)	0.335*	0.535**	0.874**	–			
Heavy exercise (days/week)	0.111	0.371*	0.324*	0.440**	–		
Heavy exercise (min/day)	− 0.048	− 0.186	− 0.045	− 0.050	0.433**	–	
Heavy exercise (min/week)	0.005	− 0.026	0.072	0.072	0.642**	0.885**	–

*p < 0.05, **p** ≤ **0.01

## Discussion

To the best of our knowledge, this is the first study to sample and compare Paralympic and recreational athletes to better understand how exercise is related to neuropathic pain among persons with SCI. Contrary to our hypotheses, Paralympic athletes experienced greater neuropathic pain intensity than recreational athletes. Neither pain catastrophizing, use of pain coping strategies nor positive affect and well-being moderated the relationship between athlete status and neuropathic pain intensity. The total number of minutes per day of moderate-intensity exercise over the past week were positively correlated with neuropathic pain intensity.

Research investigating the basal pain perception of routine exercisers is minimal. Studies of acute exercise demonstrate that a single bout of high-intensity exercise induces acute analgesic responses among both able-bodied individuals [40–42] and persons with SCI [10, 11]. However, results from the present study suggest that among persons with SCI, habitual high- frequency and intensity exercise (i.e., Paralympic-level training) is associated with greater neuropathic pain intensity than habitual recreational exercise. One explanation for these contrasting findings may be that frequent high-intensity exercise causes Paralympic athletes to regularly experience acute and chronic nociceptive pain [16, 43]. Paralympic athletes may have higher pain tolerances than recreational athletes [44, 45], and many believe that pain is a natural consequence of Paralympic sport [16]. Paralympic athletes may continue to exercise in the presence of neuropathic pain, and subsequently experience further pain. Future controlled experiments should investigate high- versus moderate frequency and intensity exercise protocols among Paralympic and recreational athletes, while assessing pain modulatory capacities to better understand the impact of varying exercise prescriptions on neuropathic pain among physically active persons with SCI.

Paralympic athletes experienced greater neuropathic pain than recreational athletes, but neither pain catastrophizing nor use of pain coping strategies significantly differed between the two groups. Additionally, neither of these psychosocial variables moderated the relationship between athlete status and neuropathic pain intensity. Coping with neuropathic pain has been reported to be one of the most difficult consequences of SCI [46–48]. Although research involving able-bodied participants suggests that elite-level athletes often develop more efficient pain coping skills than recreational athletes due to their repetitive exposure to brief periods of intense pain [45, 49], the majority of research evidence has focused on coping with musculoskeletal pain rather than neuropathic pain. Our study may have failed to detect between-groups differences in coping because athletes use different coping skills for managing neuropathic pain that are not captured by the PCS and CSQ. For instance, athletes may have exercise-related coping skills (e.g., team/social support) that impact their ability to deal with neuropathic pain sensations. To better understand how pain catastrophizing and use of coping skills impact neuropathic pain among physically active individuals with SCI, researchers must develop and implement targeted assessment tools that are informed by athletes or exercisers with SCI who experience neuropathic pain.

Despite experiencing greater neuropathic pain intensity, Paralympic athletes reported significantly greater positive affect and well-being than recreational athletes. These results support preliminary data, which suggest that Paralympic athletes with SCI have greater positive affect and well-being than non-athletes [50]. Importantly, positive affect and well-being did not *moderate* the relationship between athlete status and neuropathic pain intensity. For moderation, affect and well-being would need to be independent of exercise and pain. A recent scoping review, however, suggested that exercise concomitantly impacts both pain and well-being among persons with SCI [51], and exercise-related changes in pain may mediate changes in well-being [52]. Psychosocial factors have been shown to be more closely associated to the experience of neuropathic pain among persons with SCI than physiological factors [53]. Thus, future research should focus on investigating psychosocial moderating variables (e.g., quality of social relationships, team support) to better understand why Paralympic athletes experience greater positive affect and well-being than recreational athletes, despite experiencing greater levels of neuropathic pain.

In addition to its primary objectives, this study assessed whether frequency and duration of exercise were related to neuropathic pain intensity. None of the relationships were significant for heavy-intensity exercise. For moderate-intensity exercise, more minutes per day and more minutes per week were related to greater neuropathic pain intensity. This finding conflicts with the results of a previous study showing that an acute bout of moderate-intensity exercise led to a reduction in SCI neuropathic pain [9]. As with heavy-intensity exercise, it’s possible that the effects of individual bouts versus habitual exercise are different. Alternatively, there may be a threshold for moderate-intensity exercise. Engaging in a modest amount may have null or beneficial effects on neuropathic pain sensations [9], whereas surpassing this threshold may worsen neuropathic pain. Interestingly, frequency of moderate-intensity exercise (days per week) was unrelated to pain. Thus, if moderate-intensity exercise does exacerbate neuropathic pain, the number of days per week of exercise may not matter as much as the amount of time spent exercising. We encourage researchers to continue investigating different exercise frequencies and types to identify the optimal exercise prescription for reducing and preventing neuropathic pain among persons with SCI.

This study generated new information that expands our understanding of the relationship between SCI neuropathic pain and exercise intensity, frequency, and duration. Our study has multiple strengths. First, this is the first study to comprehensively assess neuropathic pain among athletes with SCI using clinically validated instruments (i.e., ISCIPBDS, and DN-4). Second, participants in this study were physically active and likely had better cardiometabolic health compared to inactive persons within the general SCI population [24]. To the best of our knowledge, this study included the largest sample size of healthy persons with SCI among studies that investigate neuropathic pain, coping, and pain catastrophizing. Sampling a healthier segment of the SCI population may provide a deeper understanding of neuropathic pain experiences, due to fewer confounding variables (e.g., type 2 diabetes, cardiovascular disease; [46]). And third, the study was adequately powered to test clinically and practically important research questions regarding exercise and pain among people with SCI. We achieved our target sample size by recruiting participants from around the world. Given our inclusion criteria–all participants were required to experience SCI neuropathic pain and speak English, and half were required to also be training for the Paralympics–we had a very small population to draw from. Furthermore, there are only 15 Paralympic sports for persons with SCI and many countries send only one athlete with SCI per sport; we recruited approximately 75% of this Paralympic athlete population.

There are also limitations to our study. First, participants self-reported their exercise participation which can yield recall biases. However, self-report measures of exercise are superior to technological (i.e., accelerometry) measures for persons with SCI [54]. Accelerometers have limited validity and reliability in the SCI population [55], particularly for measuring resistance-exercise [56]. Second, participants reported their average neuropathic pain over the previous 7 days. Individuals often judge painful experiences based on their peak intensity and at their end, which may have influenced participants’ ability to accurately average their neuropathic pain intensity. Third, the clinical component of the DN-4 was administered over the telephone. Participants were directed how to self-assess the presence of tactile allodynia (question 4), rather than a clinician doing the assessment. Self-assessment could compromise accuracy. Fourth, physiological variables that may explain exercise-induced effects on neuropathic pain (e.g., inflammation, glial activation) were not measured, as we could not do in-person clinical assessments. Finally, this study was not statistically powered to test for moderation effects. The absolute population size of English-speaking Paralympians who experience SCI neuropathic pain was insufficient to achieve statistical power for testing multiple moderation effects.

## Conclusion

Taken together, the results of this study suggest that Paralympic athletes experience significantly greater neuropathic pain intensity than recreational athletes, while still reporting greater levels of positive affect and well-being. Neither pain catastrophizing, nor use of pain coping skills differed based on athletic status. To guide the development of optimal exercise prescriptions that reduce neuropathic pain among physically active persons with SCI, future research should be directed toward identifying psychosocial and physiological moderators and mediators of the relationship between exercise and neuropathic pain.

## Data Availability

The datasets generated during and/or analyzed during the current study are available from the corresponding author on reasonable request. Questionnaires and scales used in this study are freely available to use.

## Abbreviations

CSQ: Coping strategies questionnaire
DN 4: Douleur neuropathique- 4
ISCIPBDS: International spinal cord injury pain basic data set
HFHI: High-frequency, high-intensity
LTPAQ SCI: Leisure time physical activity questionnaire for people with spinal cord injury
MFMI: Moderate-frequency, moderate-intensity
PCS: Pain catastrophizing scale
PAWB: Positive affect and well-being
SCI: Spinal cord injury
